# Prevalence, aetiologies and prognosis of the symptom dizziness in primary care – a systematic review

**DOI:** 10.1186/s12875-017-0695-0

**Published:** 2018-02-20

**Authors:** Stefan Bösner, Sonja Schwarm, Paula Grevenrath, Laura Schmidt, Kaja Hörner, Dominik Beidatsch, Milena Bergmann, Annika Viniol, Annette Becker, Jörg Haasenritter

**Affiliations:** Department of General Practice / Family Medicine, University of Marburg, Karl-von-Frisch-Str, 435043 Marburg, Germany

**Keywords:** Family practice, Dizziness, Vertigo, Diagnosis, Systematic review

## Abstract

**Background:**

Dizziness is a common reason for consulting a general practitioner and there is a broad range of possible underlying aetiologies. There are few evidence-based data about prevalence, aetiology and prognosis in primary care. We aimed to conduct a systematic review of symptom-evaluating studies on prevalence, aetiology or prognosis of dizziness in primary care.

**Methods:**

We systematically searched MEDLINE and EMBASE. Two independent researchers screened titles and abstracts according to predefined criteria. We included all studies evaluating the symptoms ‘dizziness’ or ‘vertigo’ as a reason for consultation in primary care. We extracted data about study population and methodology and prevalence, aetiology and prognosis. Two raters independently judged study quality and risk of bias. We investigated the variation across studies using forest plots, I^2^ and prediction intervals. Since we anticipated a great amount of clinical and unexplained statistical heterogeneity, we provided qualitative syntheses instead of pooled estimates.

**Results:**

We identified 31 studies (22 on prevalence, 14 on aetiology and 8 on prognosis). Consultation prevalence differs between 1,0 and 15,5%. The most common aetiologies are vestibular/peripheral (5,4-42,1%), benign peripheral positional vertigo (4,3-39,5%), vestibular neuritis (0,6-24,0%), Menière’s disease (1,4-2,7%), cardiovascular disease (3,8-56,8%), neurological disease (1,4-11,4%), psychogenic (1,8-21,6%), no clear diagnosis (0,0-80,2%). While studies based on subjective patient assessment reported improvement rates from 37 to 77%, these findings could not be confirmed when applying instruments that measure symptom severity or quality of life.

**Conclusion:**

There is a broad variety of possible underlying diseases for the symptom dizziness. There exist only few methodologically sound studies concerning aetiology and prognosis of dizziness.

**Electronic supplementary material:**

The online version of this article (10.1186/s12875-017-0695-0) contains supplementary material, which is available to authorized users.

## Background

Dizziness is a common complaint and frequent reason for consultation in primary care virtually affecting every person once in his or her lifetime [1]. While dizziness is a more subjective and vague symptom referring to a sense of spatial disorientation, motion of the environment, or light headedness, vertigo (as a subcategory of dizziness) is described as an illusion of movement, either of the external world revolving around the individual or of the individual revolving in space. Both symptoms can be caused by a broad spectrum of diseases ranging from benign and self-limiting (e.g. neuritis vestibularis) to severe and potentially life-threatening causes (e.g. cardiac arrhythmias or acute cerebral-vascular events) [2].

Dizziness affects patients in many ways. It is one of the most important single symptoms with a negative influence on well-being in old age [3]. While life-threatening illness is rare, many patients with dizziness do have serious functional impairment, such as increased risks for falls, significant disability, handicap and increased incidence of symptom-related fears, anxiety or depression [4, 5].

For most patients with dizziness the general practitioner (GP) is the first contact person within the official healthcare system. In comparison to different specialists like neurologists, ENT doctors or cardiologists who would only provide a workup for selected underlying diseases, GPs are trained to identify the whole range of possible aetiologies for the symptom dizziness. Hereby it is not always necessary to make an exact diagnosis as GPs in their role as gatekeepers first of all need to identify patients with uncomplicated diseases (which are often self-limiting, needing only symptomatic relief) while not overseeing potential serious diseases, which need further investigation and at times immediate therapy. In this process, GPs base their decisions predominantly on history and clinical examination. In order to do so effectively, they need setting specific knowledge about the prevalence, possible underlying aetiologies and their respective frequencies (pre-test probabilities) and the prognosis of the respective symptom to reach an acceptable diagnostic decision. Because of already selected patients and consequently resulting differing pre-test probabilities, data generated in secondary care are not applicable for the primary care context.

There has been a recently published systematic review about health care utilization, prognosis and outcomes of vestibular disease in primary care [6]. As far as we know there are no systematic reviews summarizing current evidence concerning prevalence, aetiology and prognosis of dizziness in primary care.

We aimed to answer three questions: 1) How often do patients present with dizziness in primary care, i.e. what is the prevalence/ incidence of the symptom in this setting? 2) What are the underlying aetiologies and their respective frequencies? 3) What is the prognosis of these patients?

## Methods

### Types of studies

We performed a systematic review including symptom evaluating studies about dizziness in a primary care setting. Symptom evaluating studies are defined as studies that examine patients presenting with a defined symptom in a given health care setting. These studies seek to investigate prevalence/incidence, underlying aetiologies and prognosis for patients presenting with this symptom [7].

### Inclusion- and exclusion criteria

We included all original research articles evaluating the symptoms “dizziness” and “vertigo” as a primary or a secondary consulting reason at a primary care setting. We did not limit our search to the date of publications, patient age or study quality. All types of studies except qualitative studies, case reports and narrative reviews could be included.

The findings of the studies had to include at least one of the following estimates: prevalence or incidence of dizziness and/or vertigo, information about underlying diagnoses and/or prognoses. There was no restriction regarding to the kinds of data assessment, outcome measurement or the study quality. We excluded studies that evaluated other settings than primary care (e.g. hospital care, emergency centres, secondary care) and studies, in which patients were selected before recruitment, for example because of an increased probability for a specific underlying diagnosis.

### Search strategy

In January 2015, we did a computer-based search of the PUBMED and EMBASE databases. The authors also screened the reference list of all relevant studies (snow ball search). Studies published in English, German, Dutch, Italian, Russian, Swedish, French and Spanish were included.

We used the following search syntax for electronic searches:

We defined two main search concepts (“dizziness” and “primary care”) and combined them by “AND”. To operationalize “dizziness”, we used the terms “dizziness”, “vertigo”, “giddiness” and “light headedness” in different ways of notations (in title or abstract) OR the MESH terms “dizziness” and “vertigo”. To operationalize “primary care”, we used four different strategies and combined them by OR: (1) the terms “primary care”, “general practice” or “general practitioner” in different ways of notations in title or abstract, (2) the MESH terms “family practice”, “physicians, family”, “primary Health care” and “rural health”, (3) paper was published in a journal, which typically covers primary care research OR (4) the term “primary care” in different ways of notations appeared in the affiliation of at least the main author. The whole syntax can be found in Additional file 1.

### Selection of publications

All identified studies went through a two-step selection process. First, we screened titles and abstracts. Studies meeting all three selection criteria “original research article”, “inclusion of patients because of dizziness and/or vertigo” and “primary care setting” were applied as potential appropriate. In the next step, we analysed the full texts of the selected articles regarding to inclusion- and exclusion criteria. Reasons for exclusion were documented.

Two independent review authors (SS/PG or SS/LS for title/abstracts; SS/DB or SS/MB for full texts) did the whole selection process. Disagreements were resolved by discussion between the respective two review authors (SS, PG, LS, DB, and MB). A third review author (either JH or SB) was consulted if disagreement persisted.

### Data extraction

We extracted bibliographic data (author, publication year, title and journal), country, setting, study design, inclusion- and exclusion criteria, kind of recruitment, study population (age, gender distribution), study duration. To answer the first research question (prevalence/incidence), we registered the number of dizziness cases and the number and type of the population from which the cases descended from (e.g. number of all practice consultations or all registered patients of a practice). Furthermore, we extracted all diagnostic categories and their absolute and relative frequencies (second research question “aetiology”). Finally, we documented every kind of prognostic outcome (third research question). In addition, we differentiated in our sub-analysis, where data allowed doing so, patients with dizziness from patients suffering from vertigo.

### Quality assessment

To our knowledge, no tools exist yet to assess the risk of bias in symptom evaluating studies. Therefore, we developed a standardized tool, based on the sparse methodological literature [7, 8] and own previous experience in the area [9, 10]. Two reviewers (SS and KH) independently answered signal questions and assessed the risk of bias separately for four key domains: selection of patients and GPs, data collection and patient flow, determination of the underlying aetiology, and determination of the prognosis. The characteristic values were documented with “yes”, “no” or “unclear”. In addition, reviewers rated their concern that the selection of patients and GPs might had introduced clinical heterogeneity. A detailed description of the tool can be found in Additional file 2.

### Data analysis

Studies can use different measures to report the prevalence or incidence of the symptom in a particular setting, e.g. patients presenting with the symptom in relation to all patients presenting in a specified time period; number of consultations due to the particular symptom in relation to all consultations in a specified time period etc. Since the definition of the nominator and denominator of the frequency measure has a major influence on the results, we grouped studies according to the frequency measure and plotted the results using forest plots. To answer question 2 (aetiologies and respective frequencies), we aimed to estimate how often dizziness and/or vertigo were caused by a particular aetiology. For each study presenting data for a particular condition, we calculated the respective proportion and the 95% confidence interval using the Wilson procedure with a correction for continuity. [11] We grouped all eligible studies by underlying aetiologies and plotted the results using forest plots.

We used different measures to quantify the variability across studies: I^2^ quantifies the percentage of variation that is not due to chance [12]. Tau^2^ is an estimate of the between-study variance in a random-effects meta-analysis [12]. Note that in our case the term ‘effect’ refers to a proportion, e.g. proportion of patients with a particular condition. The interpretation of tau^2^ is not intuitive. However, it allows the calculation of prediction interval. Prediction intervals can be interpreted as a range within the “true” proportion of a future study that is similar to those included in the analysis will lie with a probability of 95% [13]. Besides the number of studies, the heterogeneity across studies determines the width of the interval.

Since we expected substantial variation across studies due to different sources (methodological heterogeneity as well as clinical heterogeneity caused by different definitions of the symptom, of underlying conditions, differences in the diagnostic and prognostic work-up, case-mix, health care system and time period), we aimed to provide only qualitative summaries instead of pooled estimates.As there was only a limited number of studies that contributed data to our third research question (prognosis), we analysed these data only descriptively.

Data analysis was done by the statistical program R 3.1.2 (R Foundation for statistical analysis, Vienna, Austria).

## Results

### Search result and study selection

**Fig. 1:**
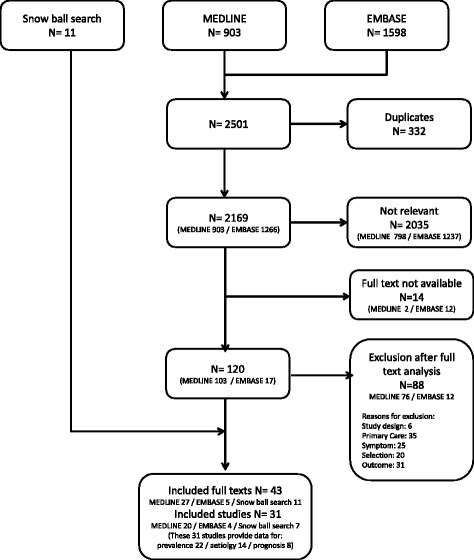
Flow chart

Our initial search identified 1598 references in EMBASE and 903 in PUBMED. The snowball search identified eleven references. After extraction of 332 duplicates, 2501 unique references remained. The title and abstract screening of these references detected 120 studies as potential appropriate. Finally, the full text analysis of these trials produced 31 studies, which met the inclusion criteria. Further details are given in a flow chart (Fig. 1).

### Included studies

Data accrue mainly from Europe (21 studies) and USA (five studies). Time of publications varied between 1972 and 2014. Twenty three studies took place in general practice, five in primary health care centres and the remaining three in hospital based primary care centres. Studies varied widely concerning the number on included patients with dizziness ranging from 30 to 10,871. The percentage of women included was mentioned in 16 studies ranging from 58 to 80%. Nineteen of the included studies recruited patients prospectively during the consultations; the other studies were based on chart review and routine data. Further details of the included studies are shown in Table 1.

**Table 1:** Brief description of the included studies

Studies	Time of recruitment	Country	Setting	Number of dizzy patients	Mean Age of study sample (range)	Female (%)	Data collection	Inclusion criteria	Exclusion criteria	Answered research questions^b^
BEACH 2014 [18]	04/2013 - 03/2014	Australia	959 general practitioners	993	n.r.	n.r.	prospective	all patients contacts ICPC: Reason for encounter vertigo/ dizziness		1
Bird 1998 [39]	08/1993 - 071995	Great Britain	3 general practices	503	median 58 (3-99)	n.r.	retrospective	complaining of symptoms allied to dizziness ➔ stratified sample was studied in more detail	patients had seen their GP about the same problem within the past 12 months	1,2
CONTENT 2007 [1]	04/2005 - 12/2006	Germany	17 general practices	607	n.r.	70.8^a^	prospective	all patients contacts ICPC: Reason for encounter N17 – vertigo/ dizziness		1
DNSGP-2 2010 [15]	01/2001 - 12/2001	Netherlands	96 family practices	3990	76.1 (SEM 0.11) (65+)	65.9	prospective	database with presented symptoms recorded as freetext: Dutch synonyms for dizziness		1,2
Ekvall 2004 [16]	1998 and 2000	Sweden	2 health care centres (14 GPs)	311	range 12-94	74.9^a^	retrospective	ICD9/10: 780.4 / R 42 and 386 / H81		1
Ekvall 2005 [3]	01/2003 - 12/2003	Sweden	6 primary health care centres	prev: 197 aet: 38	prev: range 65-99 aet: Median 83 (65-94)	prev: 70.1^a^ aet: 65.8^a^	prev: retrospective aet: prospective	prev: 65 years or older diagnosis R42 (ICD 10) aet: study participants; ICD 10 Code R 42		1,2
Fink 2007 [40]	10/1989 - 09/1999	Austria	1 general practitioner	408^a^	n.r.	n.r.	prospective	all Episodes of Cares: dizziness as reason for encounter		1
Garrigues 2008 [41]	11/2003 - 11/2004	Spain	6 primary care centres	191	55.8 (SD 17.6) (range 10+)	68.6	prospective	vertigo crisis (illusion of unequivocal rotary movement)	under 10 years	1
Gerber 1992 [42]	15 months	USA	general internal medicine group practice (4 general internists)	46	range 18+	n.r.	prospective	all physical complaints reported complaint of dizziness		1
Hanley 2002 [43]	10/1999 - 03/2000	Ireland	13 general practitioners	70	n.r.	n.r.	prospective	vertigo (do you see the world spin around you as if you had got off a playground roundabout)		2
Harding 1980 [44]	n.r.	Colombia, India, Sudan, Philippines	primary health facilities	90	range 16+	n.r.	prospective	reason for encounter of all patients aged 16 or older: dizziness	so seriously ill or required such urgent medical care that it would be unreasonable to administer the research questionnaires pat. Who refused to take part pat. Who had already attended once	1
Hopkins 1989 [45]	1981 - 1982	Great Britain	48 general practices (143 doctors)	n.r.	n.r.	n.r.	unclear	patients consulting for ICD 9: 780.4 and 386		1
Jayarajan 2003 [14]	08/2000 - 07/2001	Great Britain	53 general practitioners	62,6 average (3318)	range 5+ 5-20: 1.3% 20-40: 13.5% 40-60: 24.7% 60-80: 40.5% > 80: 20%	n.r.	retrospective	dizziness		1
Kroenke 1989 [25]	08/1984 – 07/1987	USA	Internal Medicine clinic at Brooke Army Medical Center (primary care)	55	n.r.	n.r.	retrospective	dizziness (new complaint or recurrent complaint that prompted a new diagnostic workup)	chronic dizziness	1,2,3
Kroenke 1998 [26]	n.r.	USA	Walter Reed Army Medical Center general medicine walk-in clinic	30	adults	n.r.	prospective	adult outpatients presenting with physical complaints (excl. Upper respiratory infection) dizziness		3
Kwong 2005 [4]	02/2001 – 01/2003	Canada	1 family practice center	50	range 65-91 < 80: 38% > = 80 62%	58	retrospective	ICD 9 code of “780” (dizziness) 65 years of age or older random sample of eligible charts	Patients who are discharged from service or died	2
Lawson 1999 [35]	a 3 month period	Great Britain	general practitioners from 4 practices	50	74 (61-78)	74^a^	prospective	patients presenting with dizziness more than 60 years of age		1,2
Maarsingh 2010 [23, 36]	06/2006 - 01/2008	Netherlands	45 family physicians in 24 family practices	417	78.5 (65-95)	74	prospective	dizziness being present for at least 2 weeks main reason for consultation 65 years or older	inability to speak Dutch or English, severe cognitive impairment, severe visual impairment (i.e. corrected visual acuity of less than 3/60 for the best eye), severe hearing impairment (i.e. verbal communication impossible), wheelchair dependency	2,3
Mash 2012 [46]	n.r.	South Africa	240 health workers (nurses saw 86,1% of the patients) in mobile clinics, fixed clinics and community health centres	299	n.r.	n.r.	prospective	all patients contacts ICPC: Reason for encounter N17 – vertigo/ dizziness		1
MedViP 2008 [47]	04/2001 - 12/2002	Germany	138 primary care practices	10,871	59	67.2	retrospective	ICD 10 Codes (H81, H82, A88, R42) dizziness medication synonyms for dizziness diagnoses		1,2
Morrell 1972 [48, 49]	1 year	Great Britain	1 general practice (3 doctors)	74	0-4: 1.4^a^ % 5-14: 10.8^a^ % 15-24: 17.6^a^ % 25-44: 21.6^a^ % 45-64: 25.7^a^ % > 65: 23.0^a^ %	70.3^a^	prospective	patients with a new symptom (which had not been presented to any doctor in the previous 12 months) disturbance of balance		1,2
NAMCS 1989 [50]	1981 and 1985	USA	family physicians, general practitioners and general internists	531	61.3 (25+)	66.7	prospective	all patients contacts Reason for encounter: S225.0 vertigo – dizziness		1
PCD 1994 [27, 51]	01-06 and 08-10 1991	USA	4 family group practices, 1 internal medicine group practice, 1 university family practice center, 1 solo general internist, 1 solo family physician, 1 county hospital emergency department	142	58.6 (17-90) > 60: 59.2% < 60: 40.8%	71.8	prospective	dizziness as chief complaint or part of a symptom complex that represented the principal reason for visit at least 18 years		2,3
Rieger 2014 [52]	01/2008 - 12/2008	Germany	general practitioners	489598^a^	range 18-74	n.r.	retrospective	ICD H81, H82, R42, A88.1, E53.8, F45.8, G11.8, G43.1, G45.0-, G62, G63, G90.3, H55, H83.0– 2, I95.1, N95.1 and R26 without R26.1		1
Sicras 2007 [17]	2006	Spain	5 primary health care centers	6504	n.r.	n.r.	retrospective	ICPC diagnosis N17 vertigo/ dizziness		1
Sczepanek 2011 [24]	n.r.	Germany	21 primary care practices	69	76.19 (SD 6.64) (range 65-95)	69.6	prospective	incident dizziness (less than six months) as main reason for encounter age at least 65 years	insufficient command of the German language, dementia, terminal diseases	2,3
Transition Project 2012 [53]	1995 - 2005	Netherlands, Malta, Serbia	59 general practices (69 doctors)	n.r.	n.r.	n.r.	prospective	all patients contacts ICPC: Reason for encounter N17 – vertigo/ dizziness		1
Wun 2000 [19]	12/1997 - 03/1998	China	28 commune clinics = general practice clinics (42 primary care doctors)	1331	60.8 (SD 13.5)	n.r.	prospective	all patients contacts ICPC: Reason for encounter N17 – vertigo/ dizziness		1
Yardley 1998 [22]	n.r.	Great Britain	10 general practices	aet: 143 prog: 76	aet: 59.8^a^(18+) prog: 59.6^a^	aet: 80.4^a^ prog: 82.9^a^	aet: retrospective prog: prospective	study participants 18 years and older with a complaint of dizziness patient still symptomatic	non-vestibular cause for dizziness performance of vigorous head or body movements during exercise therapy was contraindicated multiple, life-threatening or progessive central disorders	2,3
Yardley 2004 [21]	2001 - 2002	Great Britain	20 general practices	aet: 170 prog: 87	aet: 61.9^a^(18+) prog: 61.0 SD 14.42	aet: 71 prog: 71	aet: retrospective prog: prospective	study participants 18 years and older with a complaint of dizziness	Patients no longer found to be dizzy duration of dizziness less than 2 months during the past 2 years nonlabyrinthine cause of dizziness in patient records, none of the rehabilitation exercises provoked dizziness, medical contraindications for making required head movements serious comorbid conditions	2,3
Yardley 2012 [20]	10/2008 - 07/2009	Great Britain	35 general practices	112	58.2 (18 +)	75	prospective	study participants 18 years and older with a complaint of dizziness during the past two years	patients who were no longer dizzy, non vestibular causes of dizziness, dizziness was not aggravated by rapid head movements, contraindications to treatment by vestiublar rehabilitation inability to speak English	3

^a^Data not directly available in publication, but could be calculated

^b^First research question: Prevalence of the consulting reason dizziness at general practice

Second research question: Aetiology of the consulting reason dizziness at general practice

Third research question: Prognosis of the consulting reason dizziness at general practice

*n.r* Not reported, *prev* Prevalence, *aet* Aetiology, *prog* Prognosis, *BEACH* Bettering the Evaluation and Care of Health, *CONTENT* **CONT**inuous morbidity registration **E**pidemiologic **N**e**T**work, *DNSGP-2* Second Dutch National Survey of General Practice, *MedViP* Medizinische Versorgung in der Praxis, *NAMCS* National Ambulatory Medical Care Survey, *PCD* Primary Care Dizziness Study

### Quality of included studies

Quality assessment happened for four distinctive domains. For domain A (selection of patients and GPs), 14 studies had a low, three an unclear and 12 a high risk of bias. For domain B (data collection and patient flow), 19 studies had a low, one an unclear and ten a high risk of bias. For domain C (assessment of aetiology), two studies had a low, two an unclear and ten a high risk of bias. For domain D (assessment of prognosis), five studies had a low, two an unclear and one a high risk of bias. Table 2 summarizes the quality assessment of all included studies.

**Table 2:** Risk of bias

Study	Domain A: selection of patients and GPs	Domain B: Data collection and patient flow	Domain C. diagnostic work-up	Domain D: prognostic work-up
BEACH	low	low	n.r.	n.r.
Bird 1998	?	?	high	n.r.
CONTENT	low	low	n.r.	n.r.
DNSGP-2	low	low	high	n.r.
Ekvall 2004	high	high	n.r.	n.r.
Ekvall 2005	high	high	high	n.r.
Fink 2007	high	low	n.r.	n.r.
Garrigues 2008	low	low	n.r.	n.r.
Gerber 1992	high	low	n.r.	n.r.
Hanley 2002	low	low	?	n.r.
Harding 1980	low	low	n.r.	n.r.
Hopkins 1989	high	high	n.r.	n.r.
Jayarajan 2003	high	high	n.r.	n.r.
Kroenke 1989	high	high	high	high
Kroenke 1998	low	low	n.r.	?
Kwong 2005	?	high	high	n.r.
Lawson 1999 – prev.	?	low	n.r.	n.r.
*Lawson 1999 – aet.*	low	low	low	n.r.
Maarsingh 2010	low	low	low	low
Mash 2012	low	low	n.r.	n.r.
MedViP –prev.	low	high	n.r.	n.r.
*MedViP –aet.*	high	high	high	n.r.
Morrell 1972	?	low	high	n.r.
NAMCS	low	low	n.r.	n.r.
PCD	low	low	?	?
Rieger 2014	high	high	n.r.	n.r.
Sczepanek 2011	low	low	high	low
Sicras 2007	high	high	n.r.	n.r.
Transition Project	low	low	n.r.	n.r.
Wun 2000	low	low	n.r.	n.r.
Yardley 1998	high	high	high	low
Yardley 2004 – aet.	high	high	high	n.r.
*Yardley 2004 – prog.*	high	low	n.r.	low
Yardley 2012	high	low	n.r.	low

Risk of bias was rated as low, high or unclear (?)

*n.r* Not relevant, because the respective study provided no data in regard to aetiologies and/ or prognosis

prevalence, *aet* Aetiology, *prog* Prognosis

### Prevalence of the symptoms “dizziness” and “vertigo” in primary care

Twentry two studies commented on prevalence data, nine of them had a low risk of bias. Depending on numerator and denominator, results for prevalence can be grouped in four categories. Four studies describe the number of patients who have contacted a physician at least once because of dizziness/vertigo in relation to all listed patients. Prevalence ranges from 0,8% [14] to 7,9% (DNSGP-2) [15]. Seven studies describe the number of patients with dizziness in relation to all patients seen in consultation. Prevalence ranges from 1,2% [16] to 8,1% [17]. Five studies describe the number of consultations due to the symptom dizziness in relation to all consultations (e.g. if the same patient has more than one consultation he is here counted more than one time). Prevalence ranges from 1,0% (BEACH) [18] to 15,5% [19]. In four of the five studies results are below 2%, only the study done by Wun et al. represents with 15,5% an outlier. 50% of the patients seen in this study suffered from hypertension. The authors assume that patients utilized the symptom “dizziness” as a kind of entrance card to get their blood pressure monitored. Finally, four studies describe the number of consultations due to the symptom dizziness in relation to all reasons for encounter. Prevalence ranges from 0,7% (BEACH) [18] to 9,9% [19]. Forest plots and measures of heterogeneity are presented in Additional file 3.

### Aetiologies of the symptoms “dizziness” and “vertigo” in primary care

We identified 14 studies assessing data on the aetiology of dizziness and/or vertigo. The most common categories for dizziness were cardiovascular (3,8-56,8%) and otologic peripheral (5,4-42,1%) problems including Benign Positional Paroxysmal Vertigo (BPPV) and vestibular neuritis. Frequent reasons for vertigo were as well otologic peripheral causes, BPPV and vestibular neuritis, whereas cardiovascular disease did not play any role. It is noteworthy that 13 out of 14 studies used a category like “no specific diagnosis possible” and that up to 80% of cases were assigned to that category. Table 3 summarizes the results for several differential diagnoses separated for the symptoms dizziness and vertigo. Forest plots and measures of heterogeneity are presented in Additional file 4.

**Table 3:** Aetiologies of dizziness and vertigo (all studies)

Dizziness				
Aetiology	Number of studies	Number of patients	Results (range)	Heterogeneity I^2^ (95% CI)/prediction interval
Otologic peripher	10	10,658	5,4-42,1%	95.5% (93.4-96.9%) 7.7-51.6%
BPPV *(subcategory)*	6	7956	4,3-39,5%	93.2% (88.0-96.2%) 0.5-73.8%
Vestibular neuritis *(subcategory)*	6	7956	0,6-24,0%	98.4% (97.6-98.9%) 0.0-98.6%
Ménière’s disease *(subcategory)*	4	7802	1,4-2,7%	0.0% (0.0-0.0%) 1.4-2.8%
Cardiovascular	8	3011	3,8-56,8%	98.5% (98.0-98.9%) 0.4-89.1%
Neurological central	9	10,620	1,4-11,4%	78.7% (60.0-88.7%) 2.1-12.7%
Psychogenic	8	3016	1,8-21,6%	88.5% (79.7-93.5%) 1.1-31.2%
No specific diagnosis	11	10,713	0,0-80,2%	99.4% (99.3-99.5%) 1.6-90.5%
Vertigo				
Aetiology	Number of studies	Number of patients	Results (range)	Heterogeneity I^2^ /prediction interval
Otologic peripher	3	383	27.3-92.9%	96.2% (91.8-98.2%) 0.0-100.0%
BPPV *(subcategory)*	3	383	4.9-42.9%	96.4% (92.5-98.3%) 0.0-100.0%
Vestibular neuritis *(subcategory)*	3	383	8.4-40.0%	94.3% (86.8-97.6%) 0.0-100.0%
Ménière’s disease*(subcategory)*	3	383	4.2-10.0%	43.5% (0.0-83.1%) 0.0-94.1%
Cardiovascular	0			
Neurological central	1	70	4.3%	n/a
Psychogenic	1	70	1.4%	n/a
No specific diagnosis	3	383	1.4-72.7%	92.3% (80.7-96.9%) 0.0-100.0%

We could identify only two studies with a low risk of bias. Both looked at older patients with the symptom dizziness and identified cardiovascular disease as main aetiology followed by peripheral vestibular disease (see Table 4).

**Table 4:** Aetiology of dizziness (only studies with low risk of bias)

	Maarsingh 2010	Lawson 1999
Peripheral vestibular	14.4% [11.2; 18.2]	34.0% [21.6; 48.9]
BPPV		8.0% [2.6; 20.1]
Vestibular neuritis		24.0% [13.5; 38.5]
Morbus Meniere	–	2.0% [0.1-12.0]
Cardiovascular	56.8% [51.9; 61.6]^a^	48.0% [33.9; 62.4]
Neurological central	2.9% [1.6; 5.1]^b^	10.0% [3.7; 22.6]
Cerebrovascular	–	4.0% [0.7; 14.9]
Psychogenic	9.8% [7.2; 13.2]	–
Musculosceletal	3.6% [2.1; 6.0]	4.0% [0.7; 14.9]
Other internistic diseases	0.7% [0.2; 2.3]	–
Drug effects	2.4% [1.2; 4.5]	–
Other	1.2% [0.4; 2.9]	–
No specific diagnosis	8.2% [5.8; 11.3]	22.0% [12.0; 36.3]

^a^incl. Cerebrovascular; ^b^excl. cerebrovascular

### Prognosis of the symptoms “dizziness” and “vertigo” in primary care

Prognostic parameters were assessed in eight studies (two of them with a low risk of bias) using different end-points. Three studies all conducted by Yardley et al. [20–22] used the short form of the vertigo symptom scale (ranging from 0 to 60 points). All studies were intervention studies in the field of vestibular rehabilitation. In none of the studies, the respective control groups showed a significant change of 3 points (5%) at the end of the one-year follow up period.

Five studies [20–24] looked with the help of different instruments like the dizziness handicap inventory, the vertigo handicap questionnaire or the SF 36 (short version) at changes in quality of life. All studies only showed slight improvement, which was in most instances regarded as clinically non-significant.

Five studies [20, 22, 25–27] measured subjective patient assessment. Patients reported subjective improvement in al studies ranging from 37% [5] to 77% [26].

## Discussion

### Summary of principal findings

This systematic review identified 31 studies of the symptoms dizziness and/or vertigo at the primary care setting. In regard to all research questions results were very heterogeneous. The most common reasons for dizziness were cardiovascular (3,8-56,8%) and otologic peripheral problems (5,4-42,1%). Frequent reasons for vertigo were as well otologic peripheral causes, BPPV and vestibular neuritis, whereas cardiovascular disease did not play any role. In up to 80% of cases no specific diagnosis could be made. Regarding prognosis, most studies only showed slight, often non-significant improvement.

### Prevalence

Prevalence of dizziness varied markedly between studies. One reason might be that dizziness as a symptom depends on capturing and judging various sensations [2] and therefore epidemiological research faces difficulties describing and standardizing this symptom [28]. Furthermore, for identification of patients presenting with dizziness some studies counted every dizzy patient prospectively or used databases that provide data on reasons for encounter for every consultation. In contrast, other studies used routine data, with a high risk of overlooking dizzy patients with diagnoses that are not necessarily accompanied by dizziness. Both might explain heterogeneity of prevalence data.

Compared to data from emergency departments, where dizziness accounts for 2,5% [29] and 3,3% [30] of consultations, our findings provide similar figures.

### Aetiology

At primary care level GPs do not necessarily need to know the exact diagnoses. This is in accordance with studies based on routine data, where in 46,0 to 80,2% of the cases an explicit diagnosis is missing. For a symptom, that according to Sloane et al. [2] resolves for most patients spontaneously, this seems to be a reasonable approach as long as avoidable life-threading conditions can be reliably identified. On the other hand, GPs describe dizziness as a confusing and difficult problem to deal with [4]. Geser et al. [31] report that in most of the patients referred to a specialized dizziness clinic neuro-otological disorders were underdiagnosed. However, with a diagnostic approach, substantially based on medical history and clinical examination, GPs could be successfully trained to provide a better work up. Our findings support this suggestion: The proportion of unclear diagnoses decreases to 0,0-22,0%, if only prospective studies exclusively on the symptom dizziness were considered, where participating GPs presumably had to deal in one way or another with diagnostic strategies to provide a work up of their patients.

Two reviews, including studies from primary care and other settings find 4 to 48% peripheral vestibular diseases [2] and a quality-adjusted mean for peripheral vestibular diseases of 44% [32]. Studies from specialized dizziness clinics show 40,9% [33] and 46,8% [34] peripheral vestibular disease. These findings are higher than ours (5,4-42,1% peripheral vestibular diseases).

Regarding cardiovascular diseases, we found a higher proportion in older patients, especially in studies with a low risk of bias. For patients of 60 or 65 years and older the two studies with a low risk of bias showed 48,0% [35] and 56,8% ([36] cardiovascular problems. In comparison, the results of studies with a high risk of bias that included only older patients range from 10,0% [4] to 13,0% [24].

It is interesting to note that in one study conducted in the emergency department, 21,1% of dizzy patients suffered from a cardiovascular disease [30, 37], whereas in dizziness clinics cardiovascular diseases do not rank among the mentioned frequent aetiologies [31, 33, 34]. Dizziness due to cardiovascular problems might go along with other symptoms like angina pectoris that rather warrant a visit to the emergency department.

### Prognosis

Knowledge of prognosis is helpful in the context of further decision making in primary care. If dizziness in this setting has a good prognosis and will disappear in most patients from alone then it would not be necessary to always make an exact diagnosis provided red flags are taken into account. Symptomatic therapy would suffice.

While in the included studies there could be shown improvement based on subjective patient assessment, this could not be confirmed when applying instruments that measure quality of life.

In comparison, at a specialized dizziness clinic the mean score of the Dizziness Handicap Inventory (DHI) improved significantly after two year follow-up [38]. Possible explanations for this difference could be that treatment interventions in specialized clinics are more effective. Another explanation would be the shorter follow up in the primary care studies.

Overall, there is a scarcity of studies, which investigate prognosis of dizziness in primary care: Two of the four studies using the DHI include only patients of a minimum age of 65 years. The other two are intervention studies. Yardley et al. reported that about 90% of the invited patients did not participate in the study assuming that the majority of these patients were no longer dizzy [20]. This selection effect could be one explanation for the rather bad prognosis in primary care.

### Quality of included studies

The quality of the results of a systematic review depends on the quality of the included studies. Although we found 9 studies of high quality (low risk of bias for the domains A and B), which provide data on prevalence, we decided, that a meta-analysis was not reasonable because of the high clinical and methodical heterogeneity between these studies. For example, studies differ in outcomes (numerator and denominator), duration of dizziness, patient ages, proportion of women, number and type of health care institutions (practices, health care centre). Furthermore, study results depend on cultural variances, regarding, inter alia, health care systems and definition of dizziness in different countries.

Regarding aetiology we found only two studies with a low overall risk of bias (low risk of bias for the domains A, B and C). The majority of studies considers just the GPs diagnosis without follow up or standardised diagnostic approach. Moreover they do not define aetiological categories and therefore the forming of categories was difficult and at risk of overlapping. For example, some studies provided no information, under which category cerebrovascular diseases were grouped (neurological or cardiogenic).

Dizziness may have a multifactorial origin and therefore some studies allow more than one diagnosis per patient, resulting in a sum of all diagnoses over 100% and accordingly higher percentages in all aetiological categories. Other studies have a high proportion of unclear diagnosis, leading to lower percentages in other categories.

Due to the small number of trustworthy studies we could not assess, how duration of dizziness, patients age or gender influenced the proportion of underlying etiologies.

### Limitations

There are limitations to our study, influenced by three factors, which could lead to a bias in interpretation:

First, factors which influence the internal validity of the included studies like incomplete recruitment or imprecise inclusion criteria. We controlled this type of bias with rigorous quality assessment and defined clear inclusion criteria for all included studies.

Second, factors which might influence the external validity of the included studies like setting or recruitment characteristics that influence transferability to the local health care system.

Third, factors which influence the internal validity of this systematic review like mistakes in the screening process or full text analysis. Both processes were performed by two independent reviewers, which minimized this risk.

The majority of the included studies have been conducted in health care systems with a gatekeeper, some studies are from countries with direct access to specialists care. This lack of a filter might have an impact on the aetiology of the symptoms and adds an additional source of heterogeneity. These differences cannot only be seen between countries but also within one country. Germany, for example, has not an official gate keeping system but some counties have implemented a voluntary gate keeping system. Additionally, in rural areas patients tend always to contact their GP first while in urban areas specialist are contacted directly in comparatively higher frequency.

In regard to our third research question (prognosis) we could only include data looking at ‘usual care’ and did not perform an evaluation of the effectiveness of available treatments which limits the *practical application of our findings.*

Due to the missing quality standard for symptom evaluating studies we developed a more comprehensive catalogue of criteria that, when applied to the included studies, showed a broad spectrum from low to high risk of bias. As this also may introduce variance across studies, we did not pool data.

Although we have decided on structured steps concerning our search strategy, data extraction and analysis, we have not published a review protocol.

## Conclusions

In conclusion, we have shown that vertigo and dizziness are common reasons for consultation in general practice. The review identified only few studies that were pointing with a low risk of bias to the aetiology of dizziness. It would be desirable to develop better methodological procedures that allow conducting studies with a lower risk of bias. As there are already enough reliable data on prevalence, future studies should concentrate on assessing aetiology of dizziness. Many studies included in this review grouped a considerable number of patients in the category ‘diagnosis not possible’. While this surely reflects the reality in a certain percentage of patients, future research should concentrate on developing an internationally unified and accepted reference standard for the main underlying aetiologies of dizziness.

As prognosis often is not very good, rehabilitative aspects in patients with dizziness seem to be important for both research and practical implementation.

Furthermore, in many cases the underlying cause of the complaints remains unexplained. Here we can see the need of developing practical guidelines for the work up of dizziness and vertigo in primary care.

## Additional files

Additional file 1: Search strategy: contains detailed information regarding the search strategy used for this systematic review. (DOCX 14 kb)

Additional file 2: Study quality: contains detailed information on the assessment of risk of bias and sources of clinical heterogeneity. (DOCX 42 kb)

Additional file 3: Details prevalence: contains detailed information on the prevalence of dizziness. (DOCX 68 kb)

Additional file 4: Details aetiology: contains detailed information on the aetiology of dizziness. (DOCX 228 kb)

## Abbreviations

BPPV: Benign Positional Paroxysmal Vertigo
DHI: Dizziness Handicap Inventory
ENT: Ear Nose and Throat
GP: General practitioner

## References

[1] Laux G, Rosemann T, Korner T, Heiderhoff M, Schneider A, Kuhlein T, Szecsenyi J (2007). Detailed data collection regarding the utilization of medical services, morbidity, course of illness and outcomes by episode-based documentation in general practices within the CONTENT project. Gesundheitswesen.

[2] Sloane PD, Coeytaux RR, Beck RS, Dallara J (2001). Dizziness: state of the science. Ann Intern Med.

[3] Ekvall Hansson E, Mansson N-O, Hakansson A (2005). Benign paroxysmal positional vertigo among elderly patients in primary health care. Gerontology.

[4] Kwong ECK, Pimlott NJG (2005). Assessment of dizziness among older patients at a family practice clinic: a chart audit study. BMC Fam Pract.

[5] Yardley L, Owen N, Nazareth I, Luxon L (1998). Prevalence and presentation of dizziness in a general practice community sample of working age people. Br J Gen Pract.

[6] Grill E, Penger M, Kentala E (2016). Health care utilization, prognosis and outcomes of vestibular disease in primary care settings: systematic review. J Neurol.

[7] Donner-Banzhoff N, Kunz R, Rosser W (2001). Studies of symptoms in primary care. Fam Pract.

[8] Richardson WS, Wilson MC, Guyatt GH, Cook DJ, Nishikawa J (1999). Users' guides to the medical literature: XV. How to use an article about disease probability for differential diagnosis. Evidence-Based Medicine Working Group. JAMA.

[9] Viniol A, Beidatsch D, Frese T, Bergmann M, Grevenrath P, Schmidt L (2015). Studies of the symptom dyspnoea: a systematic review. BMC Fam Pract.

[10] Haasenritter J, Biroga T, Keunecke C, Becker A, Donner-Banzhoff N, Dornieden K (2015). Causes of chest pain in primary care--a systematic review and meta-analysis. Croat Med J.

[11] Newcombe RG (1998). Two-sided confidence intervals for the single proportion: comparison of seven methods. Stat Med.

[12] Higgins JPT, Green S. Cochrane Handbook for Systematic Reviews of Interventions: Version 5.1.0 [updated March 2011]. 5th ed: The Cochrane Collaboration; 2011.

[13] Higgins JPT, Thompson SG, Spiegelhalter DJ (2009). A re-evaluation of random-effects meta-analysis. J R Stat Soc Ser A Stat Soc.

[14] Jayarajan V, Rajenderkumar D (2003). A survey of dizziness management in General Practice. J Laryngol Otol.

[15] Maarsingh OR, Dros J, Schellevis FG, van Weert HC, Bindels PJ, van der Horst HE (2010). Dizziness reported by elderly patients in family practice: prevalence, incidence, and clinical characteristics. BMC Fam Pract.

[16] Ekvall, Hansson E, Mansson N-O, Hakansson A (2004). What happens with the dizzy patient in primary health care? Does education influence treatment?. Adv Physiother.

[17] Sicras-Mainar A, Velasco-Velasco S (2007). Patterns of health resources use and costs in patients with neurological disorders in primary care. [Spanish]. Rev Neurol.

[18] Britt H. General practice activity in Australia 2013–14: BEACH : bettering the evaluation and care of health. 10.33321/cdi.2003.27.68 14510069

[19] Wun Y, Lu X, Liang W, Dickinson J (2000). The work by the developing primary care team in China: a survey in two cities. Fam Pract.

[20] Yardley L, Barker F, Muller I, Turner D, Kirby S, Mullee M (2012). Clinical and cost effectiveness of booklet based vestibular rehabilitation for chronic dizziness in primary care: single blind, parallel group, pragmatic, randomised controlled trial. BMJ.

[21] Yardley L, Donovan-Hall M, Smith HE, Walsh BM, Mullee M, Bronstein AM (2004). Effectiveness of primary care-based vestibular rehabilitation for chronic dizziness. Ann Intern Med.

[22] Yardley L, Beech S, Zander L, Evans T, Weinman J (1998). A randomized controlled trial of exercise therapy for dizziness and vertigo in primary care. Br J Gen Pract.

[23] Dros J, Maarsingh OR, Beem L, van der Horst, Henriette E, ter Riet G, Schellevis FG, van Weert, Henk CPM (2012). Functional prognosis of dizziness in older adults in primary care: a prospective cohort study. J Am Geriatr Soc.

[24] Sczepanek J, Wiese B, Hummers-Pradier E, Kruschinski C (2011). Newly diagnosed incident dizziness of older patients: a follow-up study in primary care. BMC Fam Pract.

[25] Kroenke K, Mangelsdorff AD (1989). Common symptoms in ambulatory care: incidence, evaluation, therapy, and outcome. Am J Med.

[26] Kroenke K, Jackson JL (1998). Outcome in general medical patients presenting with common symptoms: a prospective study with a 2-week and a 3-month follow-up. Fam Pract.

[27] Bailey KE, Sloane PD, Mitchell M, Preisser J (1993). Which primary care patients with dizziness will develop persistent impairment?. Arch Fam Med.

[28] Grill E, Müller M, Brandt T, Jahn K (2013). Vertigo and dizziness: challenges for epidemiological research. OA Epidemiology.

[29] Kerber KA, Meurer WJ, West BT, Fendrick AM (2008). Dizziness presentations in U.S. emergency departments, 1995-2004. Acad Emerg Med.

[30] Newman-Toker DE, Hsieh Y-H, Camargo, Jr CA, Pelletier AJ, Butchy GT, Edlow JA (2008). Spectrum of dizziness visits to US emergency departments: cross-sectional analysis from a nationally representative sample. Mayo Clin Proc.

[31] Geser R, Straumann D (2012). Referral and final diagnoses of patients assessed in an academic vertigo center. Front Neurol.

[32] Kroenke K, Hoffman RM, Einstadter D (2000). How common are various causes of dizziness? A critical review. South Med J.

[33] Obermann M (2013). Chronischer Schwindel aus neurologischer Sicht. Dtsch Med Wochenschr.

[34] Strupp M, Dieterich M, Brandt T (2013). The treatment and natural course of peripheral and central vertigo. Dtsch Arztebl Int.

[35] Lawson J, Fitzgerald J, Birchall J, Aldren CP, Kenny RA (1999). Diagnosis of geriatric patients with severe dizziness. J Am Geriatr Soc.

[36] Maarsingh OR, Dros J, Schellevis FG, van Weert HC, van der Windt DA, ter Riet G, van der Horst HE (2010). Causes of persistent dizziness in elderly patients in primary care. Ann Fam Med.

[37] Moller H-J, Langer S, Schmauss M (2007). Escitalopram in clinical practice: results of an open-label trial in outpatients with depression in a naturalistic setting in Germany. Pharmacopsychiatry.

[38] Obermann M, Bock E, Sabev N, Lehmann N, Weber R, Gerwig M (2015). Long-term outcome of vertigo and dizziness associated disorders following treatment in specialized tertiary care: the Dizziness and Vertigo Registry (DiVeR) Study. J Neurol.

[39] Bird JC, Beynon GJ, Prevost AT, Baguley DM (1998). An analysis of referral patterns for dizziness in the primary care setting. Br J Gen Pract.

[40] Fink W, Haidinger G (2007). Die Häufigkeit von Gesundheitsstörungen in 10 Jahren Allgemeinpraxis. Z Allg Med.

[41] Garrigues HP, Andres C, Arbaizar A, Cerdan C, Meneu V, Oltra JA (2008). Epidemiological aspects of vertigo in the general population of the Autonomic Region of Valencia, Spain. Acta Otolaryngol.

[42] Gerber PD, Barrett JE, Barrett JA, Oxman TE, Manheimer E, Smith R, Whiting RD (1992). The relationship of presenting physical complaints to depressive symptoms in primary care patients. J Gen Intern Med.

[43] Hanley K, O' Dowd T. Symptoms of vertigo in general practice: a prospective study of diagnosis. Br J Gen Pract 2002;52:809–812. PMC1316083 12392120

[44] Harding TW, de Arango, M V, Baltazar J, Climent CE, Ibrahim HH, Ladrido-Ignacio L, et al. Mental disorders in primary health care: a study of their frequency and diagnosis in four developing countries. Psychol Med 1980;10:231–241. 10.1017/s0033291700043993 7384326

[45] Hopkins A (1989). Lessons for neurologists from the United Kingdom Third National Morbidity Survey. J Neurol Neurosurg Psychiatry.

[46] Mash B, Fairall L, Adejayan O, Ikpefan O, Kumari J, Matheel S, et al. A morbidity survey of South African primary care. PLoS One. 2012; 10.1371/journal.pone.0032358. 10.1371/journal.pone.0032358 PMC3306367 22442666

[47] Kruschinski C, Kersting M, Breull A, Kochen MM, Koschack J, Hummers-Pradier E (2008). Frequency of dizziness-related diagnoses and prescriptions in a general practice database. Z Evid Fortbild Qual Gesundhwes.

[48] Morrell DC (1972). Symptom interpretation in general practice. J R Coll Gen Pract.

[49] Morrell DC, Gage HG, Robinson NA (1971). Symptoms in general practice. J R Coll Gen Pract.

[50] Sloane PD (1989). Dizziness in primary care. Results from the National Ambulatory Medical Care Survey. J Fam Pract.

[51] Sloane PD, Dallara J, Roach C, Bailey KE, Mitchell M, McNutt R (1994). Management of dizziness in primary care. J Am Board Fam Pract.

[52] Rieger A, Mansmann U, Maier W, Seitz L, Brandt T, Strupp M, Bayer O (2014). Management of patients with the cardinal symptom dizziness or vertigo. Gesundheitswesen.

[53] Soler JK, Okkes I, Oskam S, van Boven K, Zivotic P, Jevtic M (2012). An international comparative family medicine study of the Transition Project data from the Netherlands, Malta and Serbia. Is family medicine an international discipline? Comparing incidence and prevalence rates of reasons for encounter and diagnostic titles of episodes of care across populations. Fam Pract.

