# Supplementary material for: Prevalence, aetiologies and prognosis of the symptom dizziness in primary care – a systematic review
Source: BMC Fam Pract. 2018 Feb 20;19:33. doi: 10.1186/s12875-017-0695-0 (PMC5819275; doi:10.1186/s12875-017-0695-0)
Supplement: Supplementary file 1 — Search strategy: contains detailed information regarding the search strategy used for this systematic review. (DOCX 14 kb) [file 12875_2017_695_MOESM1_ESM.docx]

# Appendix 1: Detailled search strategy

| Symptom | Symptom in all possible wordings in title and/or abstract | dizziness [TIAB]  dizzyness [TIAB]  vertigo [TIAB]  giddiness [TIAB]  light-headedness [TIAB]  lightheadedness [TIAB]  "light headedness" [TIAB]  dizzy [TIAB]  vertiginous [TIAB] | OR | |
| --- | --- | --- | --- | --- |
|  | OR | | | |
|  | Symptom as Mesh term | Vertigo [Mesh]  Dizziness [Mesh] | OR | |
| AND | | | | |
| Primary Care | Term ‘Primary Care’ in all possible wordings in title and/or abstract | “general practitioner” [TIAB]  “general practitioners” [TIAB]  “general practice” [TIAB]  “family practice” [TIAB]  “family practitioners” [TIAB]  “family practitioner” [TIAB]  “family medicine” [TIAB]  “family physician” [TIAB]  “family physicians” [TIAB]  “family doctor” [TIAB]  “family doctors” [TIAB]  “primary care” [TIAB]  “family practices” [TIAB]  “GP” [TIAB]  “GPs” [TIAB]  “GPs’” [TIAB]  “GP’s” [TIAB] | | OR |
|  | OR | | | |
|  | in one primary care relevant journal | “BMC Fam Pract” [TA]  “Fam Pract” [TA]  “J Fam Pract” [TA]  “Fam Pract Res J” [TA]  “J Am Board Fam Pract” [TA]  “Br j gen pract” [TA]  “J R Coll Gen Pract” [TA]  “J Coll Gen Pract” [TA]  “J Coll Gen Pract Res Newsl”[TA]  “Can fam physician” [TA]  “Ann Fam Med” [TA]  “Aust fam physician” [TA]  “Scand J Prim Health Care” [TA]  “Eur J Gen Pract” [TA]  “Arch Fam Med” [TA]  “J Gen Intern Med”[TA]  “Aten Primaria” [TA] | | OR |
|  | OR | | | |
|  | Term ‘Primary Care’ (different wordings) in mail address or name of institute of the author | “general practice” [AD]  “family practice” [AD]  “family medicine” [AD]  “primary care” [AD]  „community“ [AD] | | OR |
|  | OR | | | |
|  | Primary Care as Mesh term | “General Practitioners” [Mesh]  “Family Practice” [Mesh]  “Physicians, Family” [Mesh]  “Primary Health Care” [Mesh]  “Physicians, Primary Care” [Mesh]  “General Practice” [Mesh]  “Community Health Services” [Mesh]  “Rural Health Services” [Mesh]  “Rural Health” [Mesh] | | OR |
| Limits:  Publications:  NOT (editorial[pt] OR Addresses[pt] OR Bibliography[pt] OR Biography[pt] OR “Case Reports”[pt] OR Comment[pt] OR Dictionary[pt] OR Directory[pt] OR Festschrift[pt] OR “Government Publications”[pt] OR “Historical Article”[pt] OR “In Vitro”[pt] OR “Interactive Tutorial”[pt] OR Interview[pt] OR “Introductory Journal Article”[pt] OR Lectures[pt] OR “Legal Cases”[pt] OR Legislation[pt] OR News[pt] OR “Patient Education Handout”[pt] OR Portraits[pt] OR Webcasts[pt])  Languages:  AND (Dutch[lang] OR English[lang] OR French[lang] OR German[lang] OR Italian[lang] OR Russian[lang] OR Spanish[lang] OR Swedish[lang]) | | | | |
