# Supplementary material for: Prevalence, aetiologies and prognosis of the symptom dizziness in primary care – a systematic review
Source: BMC Fam Pract. 2018 Feb 20;19:33. doi: 10.1186/s12875-017-0695-0 (PMC5819275; doi:10.1186/s12875-017-0695-0)
Supplement: Supplementary file 2 — Study quality: contains detailed information on the assessment of risk of bias and sources of clinical heterogeneity. (DOCX 42 kb) [file 12875_2017_695_MOESM2_ESM.docx]

# Appendix 2: Assessment of risk of bias and sources of clinical heterogeneity

## Instrument

For each of four domain reviewers answered standardized signal questions and rated finally the risk of bias for the respective domain as low, high or unclear. Domain A and B were mandatory for all studies. Domain C and D were only eligible if the respective study reports data on research question 3 (aetiology) or research question 4 (prognosis), respectively. In these domains, it could be necessary, that signal questions had to be answered separately for each diagnostic or prognostic category, respectively.

In domain A reviewers also rated their concern that the selection of patients and/ or GPs may have introduced clinical heterogeneity.

| **Domain A: Selection of patients and GPs (refers to all studies regardless the review question)** | |
| --- | --- |
| I1 | Was the symptom to be investigated clearly described? |
| I 2 | Were the selection criteria of the patients clearly described? |
| I3 | Was a consecutive or random sample of patients enrolled? |
| I4 | Was it a multi-centre study? |
| *Judgement: Risk that the selection of patients introduced bias: low, unclear, high* | |
| I5 | Did the selection criteria of the patients permit the study population to represent the full spectrum of those presenting with the symptom in the respective setting/ addressed in the review question? |
| I6 | Were the participating health care professionals/ institutions representative for setting to be investigated in the review. |
| *Judgement: Concern that the selection of patients and GPs introduced substantial variation or clinical heterogeneity: low, unclear, high* | |
| **Domain B: Data collection and patient flow (refers to all studies regardless of the review question)** | |
| I7 | Were data about the symptom und the inclusion criteria collected directly from the patients (as opposed to a proxy like a register, routine documentation) |
| I 8 | Was the same mode of data collection used for all patients? |
| I 9 | Was the number of non-responders/ dropouts unlikely to affect the results? |
| *Judgement: Risk that the mode of data collection and/ or patient flow introduced bias: low, unclear, high* | |
| **Domain C: Determination of the underlying aetiology/ diagnostic work-up (refers only to review question “What are the underlying conditions and their respective frequencies (differential diagnosis)?”). Had to be answered for each diagnostic category separately.** | |
| I 10 | Was the etiologic category clearly defined? |
| I 11 | Was the diagnostic work up likely to correctly classify the respective aetiology? |
| I 12 | Did every patient receive the same diagnostic work up to detect the respective aetiology? |
|  | Risk that the diagnostic work up introduce bias |
| **Domain D: Determination of the prognosis/ prognostic work-up (refers only to review question “What is the prognosis of patients with the respective symptom presenting in the respective setting?”) Had to be answered for each prognostic category separately.** | |
| I 13 | Was the prognostic outcome clearly defined? |
| I 14 | Did the study design include a comparison group without the symptom? |
| I 15 | Was the work up/ measurement likely to correctly classify the respective prognostic outcome? |
| I 16 | Did every patient receive the same work up/ mode of data collection to verify the respective prognostic outcome? |
| *Judgement:* Risk that the prognostic work up introduce bias | |

## Detailed results

Domain A (Selection of patients and GPs) and domain B (Data collection and patient flow)

|  | Domain A | | | | | | | | Domain B | | | |
| --- | --- | --- | --- | --- | --- | --- | --- | --- | --- | --- | --- | --- |
| Study | I1 | I2 | I3 | I4 | Rsik of Bias | I5 | I6 | Clinical heterogeneity | I7 | I8 | I9 | Risk of bias |
| BEACH | **+** | **+** | **+** | **+** | **↓** | **+** | **+** | **↓** | **+** | **+** | **+** | **↓** |
| Bird 1998 | **+** | **-** | **+** | **-** | **?** | **+** | **+** | **↓** | **-** | **+** | **+** | **?** |
| CONTENT | **+** | **+** | **+** | **+** | **↓** | **+** | **+** | **↓** | **+** | **+** | **+** | **↓** |
| DNSGP-2 | **+** | **+** | **+** | **+** | **↓** | **-** | **+** | **↑** | **-** | **+** | **+** | **↓** |
| Ekvall 2004 | **+** | **+** | **+** | **-** | **↑** | **+** | **+** | **↓** | **-** | **+** | **+** | **↑** |
| Ekvall 2005 - prev. | **+** | **+** | **+** | **-** | **↑** | **-** | **?** | **↑** | **-** | **+** | **+** | **↑** |
| *Ekvall 2005 – aet.* | **+** | **+** | **-** | **-** | **↑** | **-** | **?** | **↑** | **+** | **+** | **-** | **↑** |
| Fink 2007 | **+** | **+** | **+** | **-** | **↑** | **+** | **+** | **↓** | **+** | **+** | **+** | **↓** |
| Garrigues 2008 | **+** | **+** | **+** | **+** | **↓** | **-** | **+** | **↑** | **+** | **+** | **+** | **↓** |
| Gerber 1992 | **+** | **+** | **?** | **-** | **↑** | **+** | **+** | **↓** | **+** | **+** | **+** | **↓** |
| Hanley 2002 | **+** | **+** | **+** | **?** | **↓** | **-** | **+** | **↑** | **+** | **+** | **+** | **↓** |
| Harding 1980 | **+** | **+** | **+** | **+** | **↓** | **+** | **?** | **↓** | **+** | **+** | **+** | **↓** |
| Hopkins 1989 | **+** | **+** | **?** | **+** | **↑** | **+** | **+** | **↓** | **-** | **+** | **+** | **↑** |
| Jayarajan 2003 | **+** | **?** | **+** | **+** | **↑** | **+** | **+** | **↓** | **-** | **+** | **?** | **↑** |
| Kroenke 1989 | **+** | **-** | **?** | **-** | **↑** | **+** | **?** | **?** | **-** | **+** | **+** | **↑** |
| Kroenke 1998 | **+** | **?** | **?** | **-** | **↓** | **+** | **?** | **↓** | **+** | **+** | **+** | **↓** |
| Kwong 2005 | **+** | **+** | **+** | **-** | **?** | **-** | **+** | **↑** | **-** | **+** | **+** | **↑** |
| Lawson 1999 – prev. | **+** | **+** | **?** | **-** | **?** | **-** | **+** | **↑** | **+** | **+** | **+** | **↓** |
| *Lawson 1999 – aet.* | **+** | **+** | **?** | **-** | **↓** | **-** | **+** | **↑** | **+** | **+** | **+** | **↓** |
| Maarsingh 2010 | **+** | **+** | **+** | **+** | **↓** | **-** | **+** | **↑** | **+** | **+** | **+** | **↓** |
| Mash 2012 | **+** | **+** | **+** | **+** | **↓** | **+** | **-** | **↓** | **+** | **+** | **+** | **↓** |
| MedViP –prev. | **+** | **+** | **+** | **+** | **↓** | **+** | **+** | **↓** | **-** | **+** | **+** | **↑** |
| *MedViP –aet.* | **+** | **+** | **+** | **+** | **↑** | **+** | **+** | **↓** | **-** | **+** | **+** | **↑** |
| Morrell 1972 | **?** | **+** | **+** | **-** | **?** | **+** | **+** | **↓** | **+** | **+** | **+** | **↓** |
| NAMCS | **+** | **?** | **+** | **+** | **↓** | **+** | **+** | **↓** | **-** | **+** | **+** | **↓** |
| PCD | **+** | **+** | **+** | **+** | **↓** | **?** | **?** | **?** | **+** | **+** | **+** | **↓** |
| Rieger 2014 | **+** | **+** | **+** | **+** | **↑** | **?** | **?** | **↑** | **-** | **+** | **+** | **↑** |
| Sczepanek 2011 | **+** | **+** | **+** | **+** | **↓** | **-** | **+** | **↑** | **+** | **+** | **+** | **↓** |
| Sicras 2007 | **+** | **+** | **+** | **-** | **↑** | **+** | **+** | **↓** | **-** | **+** | **+** | **↑** |
| Transition Project | **+** | **+** | **+** | **+** | **↓** | **+** | **+** | **↓** | **+** | **+** | **+** | **↓** |
| Wun 2000 | **+** | **+** | **+** | **+** | **↓** | **+** | **+** | **?** | **+** | **+** | **+** | **↓** |
| Yardley 1998 – aet. | **+** | **+** | **-** | **+** | **↑** | **-** | **+** | **↑** | **-** | **+** | **?** | **↑** |
| *Yardley 1998 – prog.* | **+** | **+** | **-** | **+** | **↑** | **-** | **+** | **↑** | **+** | **+** | **-** | **↑** |
| Yardley 2004 – aet. | **+** | **+** | **-** | **+** | **↑** | **-** | **?** | **↑** | **-** | **+** | **+** | **↑** |
| *Yardley 2004 – prog.* | **+** | **+** | **-** | **+** | **↑** | **-** | **?** | **↑** | **+** | **+** | **+** | **↓** |
| Yardley 2012 | **+** | **+** | **-** | **+** | **↑** | **-** | **+** | **↑** | **+** | **+** | **?** | **↓** |
| + yes, - no, ? unclear, ↑ high, ↓ low, prev. prevalence, aet. Aetiology, prog. prognosis | | | | | | | | | | | | |

Domain C (Determination of underlying aetiology, diagnostic work-up)

| **Study**  Diagnostic categories | **I10** | **I11** | **I12** | **Risk of bias** |
| --- | --- | --- | --- | --- |
| **Bird 1998** |  | - | **-** | **↑** |
| ENT, neurological, cardiac, general medical, iatrogenic, psychiatric, gynaecological, rheumatological | **-** |  |  |  |
| **DNSGP -2** |  | **-** | **-** | **↑** |
| N17 vertigo / dizziness; A06 fainting / syncope; A04 general weakness / tiredness; musculoskeletal conditions; infection; metabolic or endocrine conditions; neurologic conditions (excluding cerebrovascular conditions) | **-** |  |  |  |
| K89 Transient cerebral ischemia; K88 postural hypotension; K90 stroke / cerebrovascular accident; K86 hypertension uncomplicated; K78 atrial fibrillation / flutter; P01 feeling anxious / nervous / tense; A-Z26/A-Z27 fear of disease; R98 hyperventilation syndrome; P76 depressive disorder; adverse effect medical agent | **+** |  |  |  |
| cardiovascular conditions; peripheral vestibular disease; psychiatric conditions | **?** |  |  |  |
| **Ekvall 2005** |  |  |  | **↑** |
| BPPV | **+** | **+** | **+** |  |
| vestibular neuronitis | **+** | **-** | **?** |  |
| multisensory dizziness with age as one factor | **-** | **-** | **?** |  |
| **Hanley 2002** |  | **?** | **?** | **?** |
| psychological origion | **-** |  |  |  |
| benign positional vertigo, acute vestibular neuronitis, Meniere’s disease, vascular origin (TIA, stroke), neurological origin (MS) | **+** |  |  |  |
| **Kroenke 1989** |  | **?** | **+** | **↑** |
| organci, psychologic | **-** |  |  |  |
| **Kwong 2005** |  | **-** | **-** | **↑** |
| BPV, labyrinthitis, TIA/ stroke, hypertension, depression/ anxiety, arrhythmia, alcohol, dehydration | **+** |  |  |  |
| **Lawson 1999** |  | **+** | **?** | **↓** |
| cardiovascular diagnoses (total cardial sinus hypersensitivity (CSH) or in combination; vasodepressor carotid sinus hypersensitivity, cardioinhibitory carotid sinus hypersensitivity, mixed carotid hypersensitivity, Vasovagal, vasovagal syncope (VV) + VDCSH, VV + orthostatic hypotension (OH) + VDCSH, Arrhythmia, orthostatic hypotension, Mixed CSH + VV)  peripheral vestibular disorders (vestibular neuronitis, BPPV, Meniere)  central neurological disorders (severe cervical spondylosis, drop attacks, stroke disease, Migraine, bilateral carotid stenosis (>90%)) | **+** |  |  |  |
| **Maarsingh 2010** |  | **+** | **+** | **↓** |
| cardiovaskular disease (incl. cerebrovascular); locomotor disease; metabolic or endocrine; neurological (excl. cereborvascular); psychiatric; periphereal vestibular disease | **-** |  |  |  |
| adverse drug effect | **+** |  |  |  |
| impaired vision | **?** |  |  |  |
| **MedViP** |  | **-** | **-** | **↑** |
| R42 dizziness and giddiness | **-** |  |  |  |
| H81. Ménière disease, H81.1 benign paroxysmal vertigo, H 81.2 vestibular neuronitis | **+** |  |  |  |
| H81.3 other peripheral vertigo, H 81.4 vertigo of central origin, A88.1 epidemic vertigo, H81.8 other disorders of vestibular function, H 81.9 disorder of vestibular function, unspecified | **?** |  |  |  |
| **Morrell 1972** |  | **-** | **-** | **↑** |
| symptomatic diagnosis (vertigo), psychiatric disorder | **-** |  |  |  |
| benign hypertension, Meniere, motion sickness, wax in the ears | **+** |  |  |  |
| vascular lesion oft he CNS | **?** |  |  |  |
| **PCD** |  | **?** | **-** | **?** |
| otologic, cardiovascular, psychiatric, neurological, infectious, metabolic or endocrine | **-** |  |  |  |
| labyrinthitis, BPPV, presyncope, hypertension, anxiety, hyperventilation, TIA, otitis media, sinusitis, anaemia, dehydration, drug intoxicity | **+** |  |  |  |
| **Sczepanek 2011** |  | **-** | **-** | **↑** |
| multicausal, cardiogenic, cervicogenic, symptomatic, peripheral verstibular, psychogenic, central | **-** |  |  |  |
| BPPV, vestibular neuritis, Meniere | **+** |  |  |  |
| **Yardley 1998** |  | **-** | **-** | **↑** |
| ear disease, vertigo | **-** |  |  |  |
| Meniere, labyrinthitis, BPPV | **+** |  |  |  |
| aural surgery | **?** |  |  |  |
| **Yardley 2004** |  | **-** | **-** | **↑** |
| Meniere disease, labyrinthitis, benign positional vertigo, vestibular neuronitis | **+** |  |  |  |
| vestibular imbalance or disorder, otologic disorder | **-** |  |  |  |
| **+ yes, - no, ? unclear, ↑ high, ↓ low, prev. prevalence, aet. aetiology, prog. prognosis** | | | | |

Domain D (Determination of prognosis, prognostic work-up)

| **Study**  Prognostic categories | **I13** | **I14** | **I15** | **I16** | **Risk of bias** |
| --- | --- | --- | --- | --- | --- |
| **Kroenke 1989** |  |  |  |  |  |
| outcome (patient record) | **?** | **-** | **-** | **?** | **↑** |
| **Kroenke 1998** |  |  |  |  |  |
| subjective improvement | **?** | **-** | **?** | **+** | **?** |
| **Maarsingh 2010** |  |  |  |  |  |
| DHI | **+** | **-** | **+** | **+** | **↓** |
| **PCD** |  |  |  |  |  |
| subjective improvement | **+** | **-** | **?** | **+** | **?** |
| **Sczepanek 2011** |  |  |  |  |  |
| DHI, DiNA, SF-12, ADL | **+** | **-** | **+** | **+** | **↓** |
| GDS | **+** | **-** | **-** | **+** | **↑** |
| **Yardley 1998** |  |  |  |  |  |
| Vertigo Symptom Scale, Vertigo Handicap Questionaire | **+** | **-** | **+** | **+** | **↓** |
| subjective improvement | **+** | **-** | **?** | **+** | **?** |
| HADS, Romberg, provocative movements, Dizziness Belief Scale | **+** | **-** | **-** | **+** | **↑** |
| **Yardley 2004** |  |  |  |  |  |
| Vertigo Symptom Scale, DHI, SF-36 | **+** | **-** | **+** | **+** | **↓** |
| HADS, Balance Performance Monitor, provoked symptoms | **+** | **-** | **-** | **+** | **↑** |
| **Yardley 2012** |  |  |  |  |  |
| Vertigo Symptom Scale, DHI, EuroQol EQ-5D | **+** | **-** | **+** | **+** | **↓** |
| subjective improvement | **+** | **-** | **?** | **+** | **?** |
| HADS | **+** | **-** | **-** | **+** | **↑** |
