# Supplementary material for: Prevalence, aetiologies and prognosis of the symptom dizziness in primary care – a systematic review
Source: BMC Fam Pract. 2018 Feb 20;19:33. doi: 10.1186/s12875-017-0695-0 (PMC5819275; doi:10.1186/s12875-017-0695-0)
Supplement: Supplementary file 3 — Details prevalence: contains detailed information on the prevalence of dizziness. (DOCX 68 kb) [file 12875_2017_695_MOESM3_ESM.docx]

# Appendix 3: prevalence of dizziness – detailed information

| **Patients with dizziness – Patients registered at the practices** | | |
| --- | --- | --- |
| 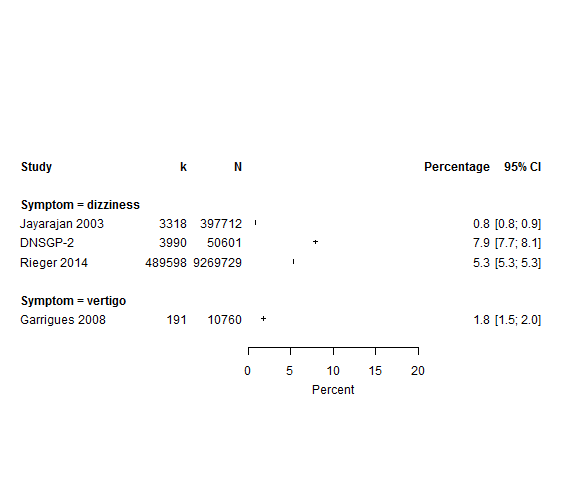 | | |
| I² | Tau² | Prediction interval |
| 100.0% (100.0-100.0%) | 0.893 | 0.0-73.4% |
| k: number of patients consulting at least once because of dizziness/ vertigo. N: number of all patients registered at the practices. | | |

Garrigues 2008 included only patients with vertigo crisis.

| **Patients with dizziness – Patients consulting the GPs / practices** | | |
| --- | --- | --- |
| 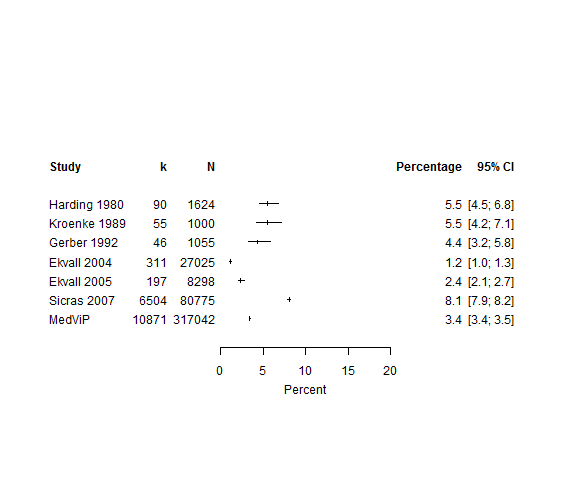 | | |
| I² | Tau² | Prediction interval |
| 99.8% (99.8-99.9%) | 0.442 | 0.6-19.5% |
| k: number of patients consulting at least once because of dizziness. N: number of all patients consulting the GPs / practices | | |

| **Consultations because of dizziness – All consultations** | | |
| --- | --- | --- |
| 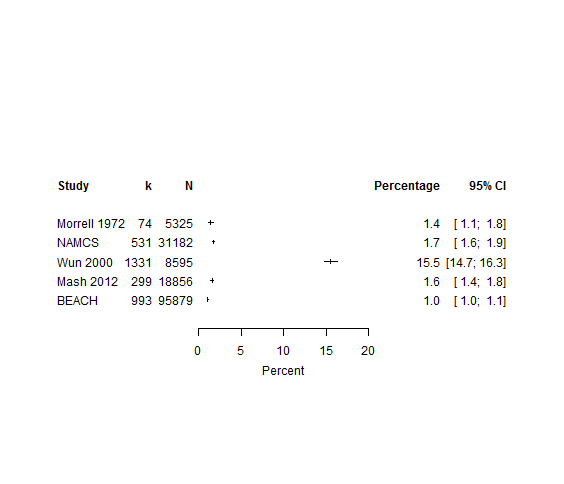 | | |
| I² | Tau² | Prediction interval |
| 99.9% (99.9-99.9%) | 2.35 | 0.0-83.3% |
| k: number of consultations because of dizziness/ reason for encounter = dizziness. N: number of all consultations. | | |

| **Reason for encounter = dizziness – All reasons for encounter** | | |
| --- | --- | --- |
| 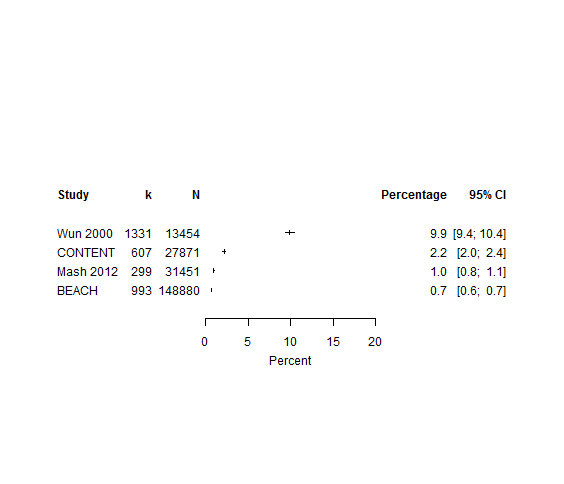 | | |
| I² | Tau² | Prediction interval |
| 99.9% (99.9-99.9%) | 2.142 | 0.0-95.8% |
| k: Reason for encounter = dizziness. N: All reasons for encounter. | | |
