# Supplementary material for: Prevalence, aetiologies and prognosis of the symptom dizziness in primary care – a systematic review
Source: BMC Fam Pract. 2018 Feb 20;19:33. doi: 10.1186/s12875-017-0695-0 (PMC5819275; doi:10.1186/s12875-017-0695-0)
Supplement: Supplementary file 4 — Details aetiology: contains detailed information on the aetiology of dizziness. (DOCX 228 kb) [file 12875_2017_695_MOESM4_ESM.docx]

# Appendix 4: Aetiology of dizziness – detailed information

| Aetiology: otologic peripher | | |
| --- | --- | --- |
| 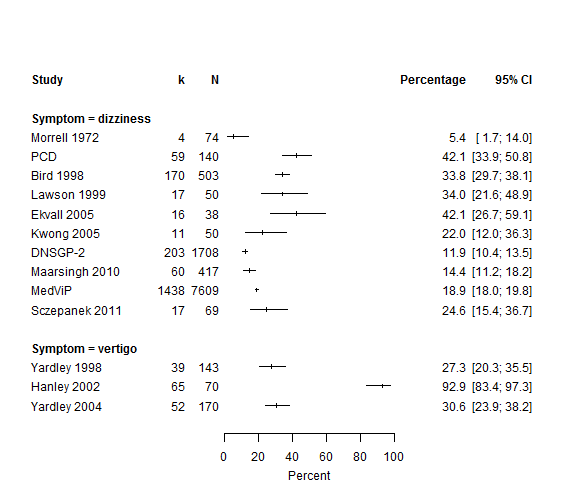 | | |
| I² | Tau² | Prediction interval |
| 95.9% (94.3-97.0%) | 0.347 | 9.1-60.1% |
| k: number of patients with the respective aetiology. N: number of all patients presenting with dizziness / vertigo | | |

| Aetiology: BPPV | | |
| --- | --- | --- |
| 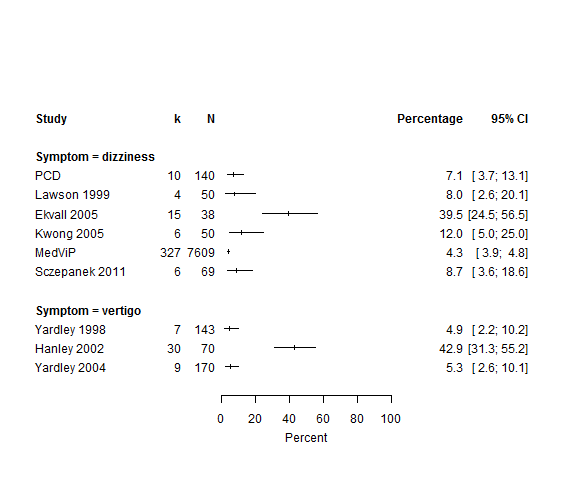 | | |
| I² | Tau² | Prediction interval |
| 95.8% (93.8-97.2%) | 1.573 | 0.5-73.7% |
| k: number of patients with the respective aetiology. N: number of all patients presenting with dizziness / vertigo | | |

| Aetiology: vestibular neuritis | | |
| --- | --- | --- |
| 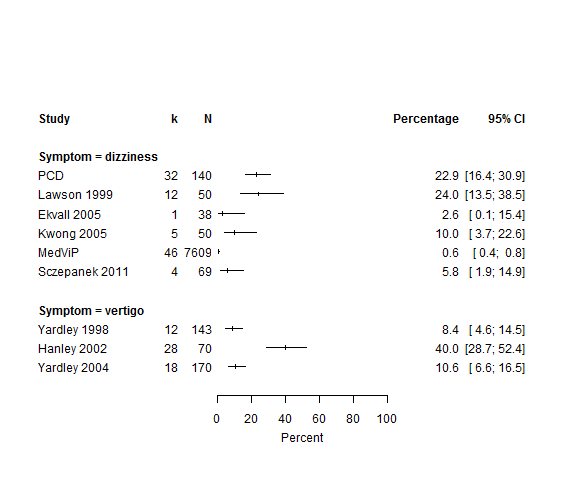 | | |
| I² | Tau² | Prediction interval |
| 98.2% (97.5-98.6%) | 4.025 | 0.1-93.7% |
| k: number of patients with the respective aetiology. N: number of all patients presenting with dizziness / vertigo | | |
| Aetiology: Ménière's disease | | |
| 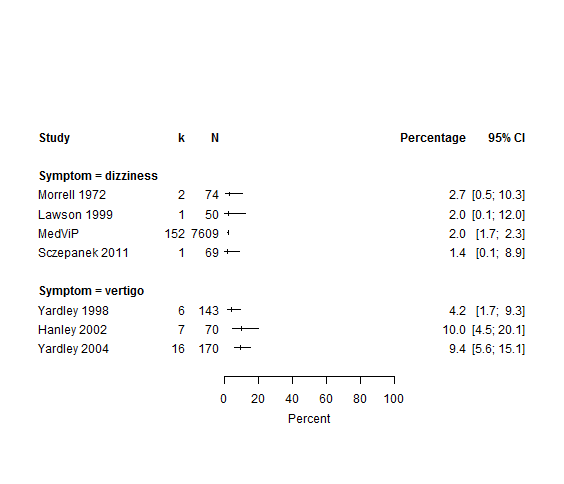 | | |
| I² | Tau² | Prediction interval |
| 88.4% (78.5-93.7%) | 0.845 | 0.3-35.4% |
| k: number of patients with the respective aetiology. N: number of all patients presenting with dizziness / vertigo | | |

| Aetiology: cardiovascular | | |
| --- | --- | --- |
| 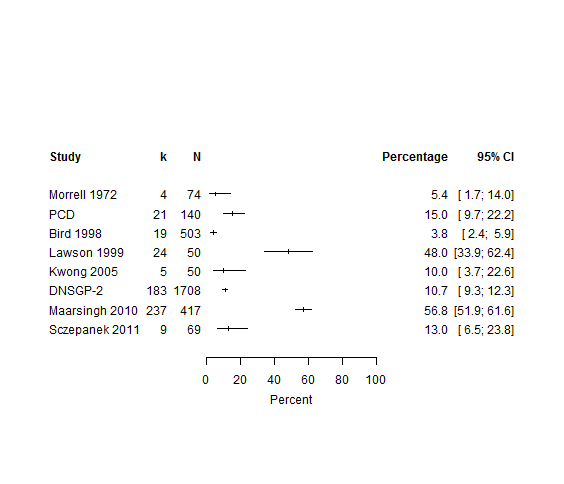 | | |
| I² | Tau² | Prediction interval |
| 98.5% (98.0-98.9%) | 2.148 | 0.4-89.1% |
| k: number of patients with the respective aetiology. N: number of all patients presenting with dizziness / vertigo | | |

| Aetiology: neurological central | | |
| --- | --- | --- |
| 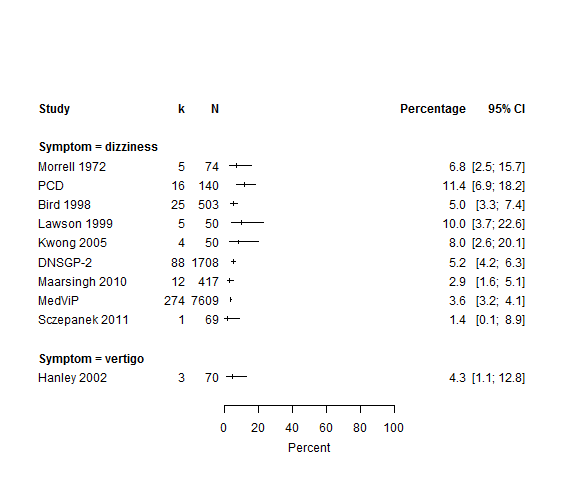 | | |
| I² | Tau² | Prediction interval |
| 76.1% (55.7-87.1%) | 0.127 | 2.2-12.0% |
| k: number of patients with the respective aetiology. N: number of all patients presenting with dizziness / vertigo | | |

| Aetiology: cerebrovascular | | |
| --- | --- | --- |
| 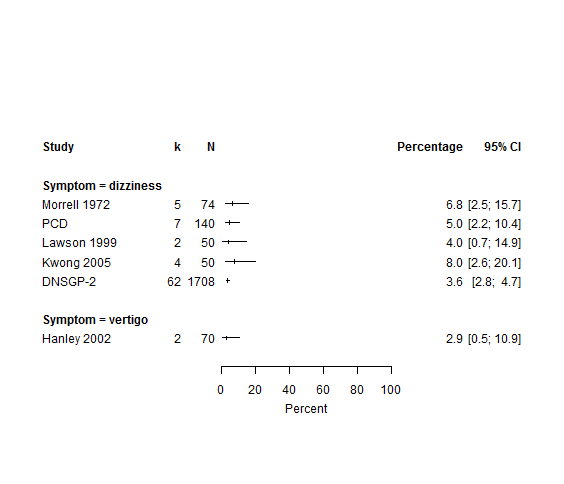 | | |
| I² | Tau² | Prediction interval |
| 0.0% (0.0-72.6%) | 0 | 3.0-5.4% |
| k: number of patients with the respective aetiology. N: number of all patients presenting with dizziness / vertigo | | |

| Aetiology: psychogenic | | |
| --- | --- | --- |
| 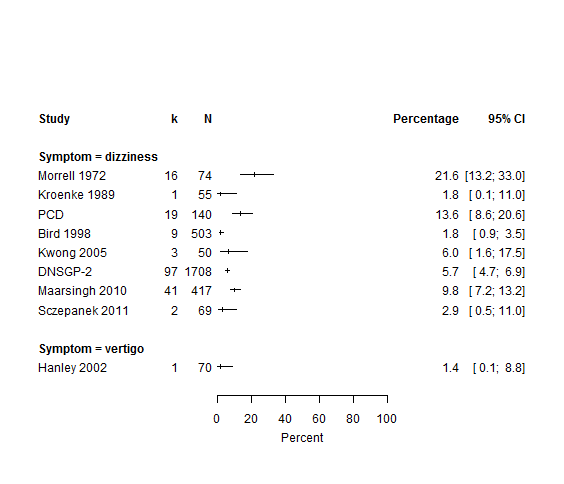 | | |
| I² | Tau² | Prediction interval |
| 87.4% (78.3-92.7%) | 0.49 | 1.1-28.3% |
| k: number of patients with the respective aetiology. N: number of all patients presenting with dizziness / vertigo | | |

| Aetiolgoy: other internistic diseases | | |
| --- | --- | --- |
| 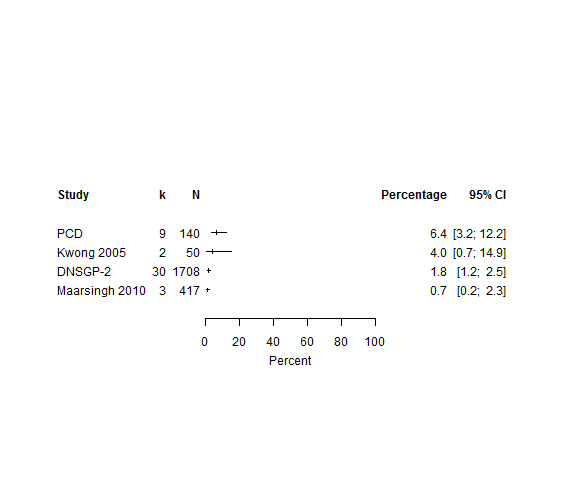 | | |
| I² | Tau² | Prediction interval |
| 81.9% (53.2-93.0%) | 0.662 | 0.0-58.7% |
| k: number of patients with the respective aetiology. N: number of all patients presenting with dizziness / vertigo | | |

| Aetiology: drug effects | | |
| --- | --- | --- |
| 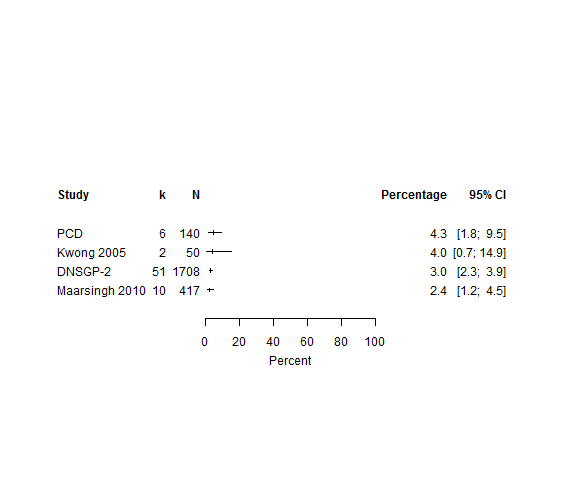 | | |
| I² | Tau² | Prediction interval |
| 0.0% (0.0-68.8%) | 0 | 1.8-5.0% |
| k: number of patients with the respective aetiology. N: number of all patients presenting with dizziness / vertigo | | |

| Aetiology: no specific diagnosis | | |
| --- | --- | --- |
| 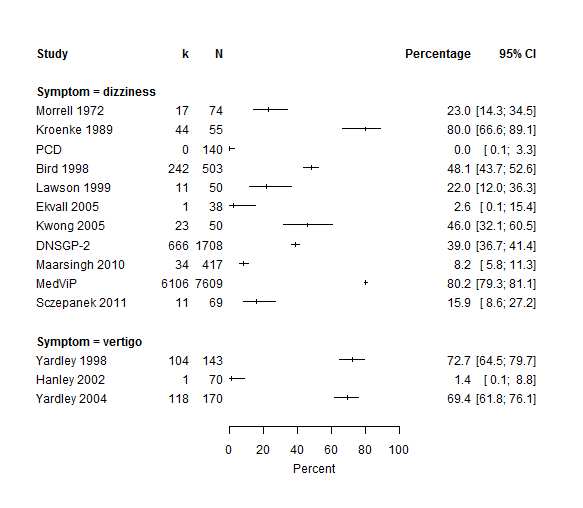 | | |
| I² | Tau² | Prediction interval |
| 99.2% (99.1-99.4%) | 1.666 | 2.4-89.3% |
| k: number of patients with the respective aetiology. N: number of all patients presenting with dizziness / vertigo | | |

| Aetiology: no specific diagnosis | | |
| --- | --- | --- |
| 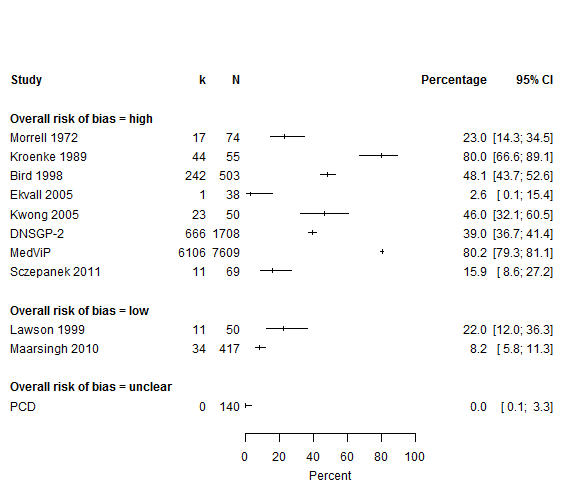 | | |
| I² | Tau² | Prediction interval |
| 99.4% (99.3-99.5%) | 1.813 | 1.6-90.5% |
| k: number of patients with the respective aetiology. N: number of all patients presenting with **dizziness** | | |
